# CCL20 secreted from IgA1-stimulated human mesangial cells recruits inflammatory Th17 cells in IgA nephropathy

**DOI:** 10.1371/journal.pone.0178352

**Published:** 2017-05-26

**Authors:** Guoyuan Lu, Xiaopan Zhang, Lei Shen, Qing Qiao, Yuan Li, Jieqiong Sun, Jinping Zhang

**Affiliations:** 1 Department of Internal Medicine, Division of Nephrology, the First Affiliated Hospital of Soochow University, Suzhou, Jiangsu Province, People’s Republic of China; 2 Institutes of Biology and Medical Sciences, Soochow University, Suzhou, Jiangsu Province, People’s Republic of China; INSERM1163, FRANCE

## Abstract

IgA nephropathy (IgAN) is the most common primary glomerulonephritis characterized by human mesangial cells (HMC) proliferation and extracellular matrix expansion associated with immune deposits consisting of galactose-deficient IgA1. However, how IgA1 contributes to IgAN has yet to be completely elucidated. In this study, the expression profile of chemokines was more altered in IgA1-treated HMC than in the control group. CCL20 was significantly higher either in the serum of IgAN patients or in IgA1-treated HMC. Further experiments demonstrated that CCR6, the only receptor of CCL20, was highly expressed in activated T cells. Intracellular staining assay and cytokine expression profile implied that CCR6^+^ T cells produced high IL-17 levels. Transwell experiment immunohistochemistry and immunofluorescence experiments extensively demonstrated that CCL20 could recruit inflammatory Th17 cells to the kidneys. These phenomena caused a series of immune inflammatory responses and further damaged the kidneys. Therefore, HMC stimulated by IgA1 could produce CCL20 and consequently recruit inflammatory Th17 cells to the kidneys to induce further lesion in IgA nephropathy.

## Introduction

IgA nephropathy (IgAN), which is also known as Berger’s disease, was first described and named by the French scholar Berger in 1968 [1]. Galactose-deficient IgA1 deposits in the glomerular mesangium and manifests the clinical presence of microscopic or macroscopic hematuria. Approximately 20% to 30% of IgAN cases have resulted in chronic renal failure (CRF) for the past 20 years [2]. It is one of the most common causes of chronic kidney damage causing primary glomerulonephritis. The basic abnormality of IgAN is considered the mesangial deposition of aberrant IgA1, and this abnormality trigger a series of immune inflammatory responses.

The diagnostic histological feature of IgAN is the deposition of pathogenic IgA1 complexes in the glomerular mesangium [3, 4], which indicates that mesangial cells are the first targets of injury [5]. Thereafter, HMC releases pro-inflammatory and pro-fibrogenic mediators, including IL-1β, IL-6, IL-8, MCP-1, and TNF-α [6–10]. The dysplasia and apoptosis of HMC are also modulated by IgA1 complexes [11–13]. The subsequent immune inflammation and cellular proliferation and apoptosis lead to the progression of IgAN. However, the mechanism by which the deposition of IgA1 in mesangial cells induces inflammation is unclear.

Chemokines and chemokine receptors are crucial factors involved in the generation and development of renal diseases, which can recruit inflammatory cell subsets under physiological and inflammatory conditions [14]. HMC produces some chemokines to induce inflammation, but whether IgA or IgA complex interacts with HMC remains unknown.

In this study, CCL20 was highly expressed in HMC cells treated with IgA1 purified from the serum of IgAN patients. ELISA revealed that the concentration of CCL20 was higher in the serum of IgAN patients than those in the serum of healthy individuals. FACS analysis demonstrated that CCR6, a CCL20 receptor, was expressed in activated T cells. CCL20 and CCR6^+^ T cells were detected in the kidney of IgAN patients through immunohistochemistry and immunofluorescence staining, respectively. These T cells produced interleukin-17 (IL-17). These results suggested that the IgA1 interacted with HMC to induce HMC and consequently produced CCL20. As a result, CCR6^+^ attracts the activated Th17 cells to the kidney and contributed to renal inflammation.

## Materials and methods

### Ethics statement

Patients and healthy controls (HC) were enrolled between July 2012 and May 2016 at the First Affiliated Hospital of Soochow University, China. The study was approved by the First Affiliated Hospital of Soochow University Research Ethics Committee, and all of the subjects provided their written informed consent for sample collection.

### Sample collection

All of the patients were diagnosed by obtaining their renal biopsy between July 2012 and May 2016 at the First Affiliated Hospital of Soochow University, China. A total of 183 patients (87 males and 96 females aged 18 to 65 years with a mean age of 33.5±9.17 years) with IgAN had some renal function. Patients with IgAN and severe renal damage (over Hass III and/or serum creatinine >450 μmol/L) were excluded from the study. Non-IgAN patients, such as those with renal cancer, nephrotic syndrome, lupus nephritis, and diabetic nephropathy, were evaluated through renal biopsy and were included as control patients for immunofluorescence and immunohistochemistry. As a healthy control population, who were comparable in terms of age and geographical origin, 218 gender- and age-matched subjects (105 males and 113 females) aged 26 to 68 years (mean age = 46±7.2 years) from our hospital were recruited. At the time of blood collection and renal biopsy, the subjects with fever or respiratory, gastrointestinal tract, or urinary infections were excluded. In addition, 61 IgAN patients were assigned for serum IgA1 purification and incubated with HMC, while the other patients were subjected to ELISA. A total of 57 HC were delegated for IgA1 isolation, 53 HC were used for plasma cytokine examinations, and the remaining HC were utilized for screening peripheral blood mononuclear cells (PBMC) or T cells.

The randomly collected histological section samples were used for immunohistochemistry and immunofluorescence from patients with IgAN and other renal diseases, including renal cancer, non-IgAN nephrotic syndrome, and lupus nephritis, as controls. Para-carcinoma mainly refers to kidney tissues of more than 5 cm in size. The detailed information of the experimental objects is listed in Table 1 (Data are expressed as the mean ±SD; RBC/HPF: red blood cells per high power field; g/24H:24 hours Proteinuria quantitative).

**Table 1:** Patients information in experiments.

	Total (n =)	Age	Male/Female	Serum creatinine	hematuria	Proteinuria
		year	M:F	mg/dl	RBC/HPF	g/24H
IgA Nephropathy	46	33±10.8	21/25	71±12.1	63±163.3	1.4±1.2
Rental cancer (para-carcinoma)	16	49±15.7	10/6	75±15.2	18±24.1	not test
Nephrotic syndrome (except IgAN)	12	37±14.3	4/7	82±22.9	25±33.6	3.1±2.2
Lupus nephritis	2	25	0/2	123.5	59.5	2.3
Diabetic nephropathy	1	39	1/0	61	7	2.6

### Antibodies

The following antibodies were purchased from Biolegend company (San Diego, California, America): anti-CD3-APC (HIT3a), anti-CD3-PE(OKT32), anti-CD3-Biotin (SK7), anti-CD28-LEAF^TM^ Purified (CD28.2), anti-CD11c-PE(3.9), anti-CD11b-PerCP (M1/70), anti-CD196(CCR6)-APC (G034E3), anti-CD196(CCR6)-FITC (G034E3), anti-IFN-γ-APC (4S.B3), anti-IL-17A-PE (BL168), Anti-CD44-FITC (BJ18), and anti-IL-4-PE (8D4-8). The HRP-labeled anti-Human IgG antibody (H&L) was purchased from GenScript (Nanjing, China). The anti-Human IgA1 antibody (B3506B4) was procured from Abcam company (Cambridge, England). The anti-CCL20-Biotinylated antibody (BAF360) and anti-CCL20 antibody (67310) were obtained from R&D Systems (Minnesota, USA).

### HMC culture

HMC were purchased at passage 3 (BioHemes, China). HMC were grown in Dulbecco’s modified Eagle’s medium containing 10% fetal bovine serum (FBS), penicillin (100 U/mL) and streptomycin (100 U/mL) at 37°C in an atmosphere of 5%CO_2_/95% air. Cells grown at passages 5 to 8 were used in all experiments. HMC were respectively incubated with 100 μl/mL serum or 100 μg/mL IgA1 from IgAN and HC for 48 h or 24 h. Then, cells were collected and RNA was extracted for further experiments.

### Real-time PCR assay

Total cellular RNAs were all extracted using RNAiso Plus reagent (Takara Biotechnology Co., Ltd) from the HMC by different treatments. To detect the expression of CCL20, RNAs were reversed to cDNA by using the M-MLV reverse transcriptase with Oligo (dT)s. Quantitative real-time PCR was performed in triplicate by using FastStart Universal SYBR Green Master (Roche) on an Eppendorf Real-Time Detection System. Moreover, the gene was normalized to the amount of GAPDH mRNA expression. The RT-PCR primers of the genes are listed in Table 2.

**Table 2:** Primers used for real-time PCR.

Gene	Primer pairs	Sequence(5equence)
CCL20	Forward	GCGAATCAGAAGCAGCAAGC
	Reverse	GGATTTGCGCACACAGACAA
IL-2	Forward	AGTGCACCTA CTTCAAGTTCT
	Reverse	AATGTGAGCA TCCTGGTGAG
IL-4	Forward	CAGCCTCACAGAGCAGAAG
	Reverse	CTTCTCATGGTGGCTGTAGA
IL-5	Forward	TCTGAGGATT CCTGTTCCTG
	Reverse	GAATTGGTTTACTCTCCGTCT
IL-6	Forward	GAGTAACATGTGTGAAAGCAG
	Reverse	GCTTGTTCCTCACTACTCTC
IL-8	Forward	CTGAGAGTGATTGAGAGTGG
	Reverse	CAACCCTCTGCACCCAGTT
IL-9	Forward	CTCAGATGACCAATACCACC
	Reverse	GACTCTTCAGAAATGTCAGCG
IL-10	Forward	CCAGTCTGAGAACAGCTGC
	Reverse	GGATCATCTCAGACAAGGCT
IL-17	Forward	GATGGTCAAC CTGAACATCC
	Reverse	CGTTGATGC AGCCCAAGTG
IL-21	Forward	GCCACGGCACAGTCATTGAAA
	Reverse	ACATGAAGGGCATGTTAGTCT
IL-23P19	Forward	ATCTAAGAGAAGAGGGAGATG
	Reverse	ATCCGATCCTAGCAGCTTCT
TNF-a	Forward	GACAAGCCTG TAGCCCATG
	Reverse	TACAGGCCCTCTGATGGCA
IFN-G	Forward	GCAGAGCCAAATTGTCTCCT
	Reverse	GCGTTGGACATTCAAGTCAG
Human GAPDH	Forward	AGAAGGCTGGGGCTCATTTG
	Reverse	AGGGGCCATCCACAGTCTTC

### Determination of cytokine concentration in serum

CCL20 was measured by using specific ELISA kits with commercially available paired antibodies (R&D Systems, DM3A00). Specific methods were carried out in accordance with the manufacturer’s instructions. The sensitivity of the CCL20 ELISA was 0.87 pg/mL.

### Purification of serum IgA1

IgA1 was purified with Jacalin-Agarose (InvivoGen, gel-jac-5) from IgAN patients’ and healthy subjects’ serum. First, 1 mL of immobilized Jacalin-Agarose was packed in 10 mL affinity chromatography column. Then, 5 mL of equilibration and wash buffer (10 mM sodium phosphate, 150 mM sodium chloride, pH7.2) was used to equilibrate the column. Afterward, appropriate amounts of the serum were dialyzed against 100 volumes of this buffer. The dialyzed sample was filtered with a 0.2 μm filter, loaded onto the column, and incubated at room temperature for 90 min. After all of the samples flowed down at a slow velocity, the column was washed with 20 mL of this buffer. Lastly, it was eluted with 10 mL of elution buffer (0.1 M melibiose in PBS), washed with 10 mL of regeneration buffer (20% (v/v) ethanol in PBS), and stored at 4°C. All chromatography steps were performed at a flow rate under gravity flow. The purity and concentration of IgA1 fractions were confirmed through SDS-PAGE and micro BCA protein method and stored at −20°C until use.

### Isolation of PBMC and T cells

PBMC from a healthy person was isolated from the blood by using Lympholyte®-Hypaque density gradient at room temperature following sterile techniques. In brief, human whole peripheral blood was collected in a tube containing an anticoagulant. Next, the blood was diluted with an equal volume of PBS. The 15 mL centrifuge tube was added with 3 mL Lympholyte®-H. Using as minimal mixing as possible at the interface, we carefully layered 6 mL of diluted blood over the Lympholyte®-H. At room temperature, the 15 mL tube was centrifuged for 30 min at 400 g. After that, the cells from the interface was carefully removed and transferred to a new centrifuge tube. Then, the transferred cells were washed two to three times with RPMI1640 medium without FBS or PBS before further processing. For T cell isolation, PBMC was stained with anti-CD3-Biotin antibody for 30 min, washed with PBS for three times, and stained with microBeads for at least 20 min. Lastly, T cells were purified via magnetic bead cell sorting.

### Chemotaxis assay

For the chemotaxis experiment, approximately 5×10^5^ cells/well of peripheral blood cells with or without neutrophils) were isolated from normal donors by density gradient centrifugation, suspended in RPMI1640 medium, and seeded on the upper chamber of the insert. In the lower compartment of the transwell, the recombinated-human CCL20 (rhCCL20 R&D 360-MP-025/CF) was added in the RPMI1640 medium. After 60 min, the cells in the lower compartment were harvested, and relevant antibodies were stained for flow analysis by using a flow cytometer (BD FACS Calibur). Transmigration assays were performed to elucidate the possibility that the interaction between the cytokine of CCL20 from the affected individuals and the peripheral blood cells induces transmigration. In this assay, a plain culture medium was added to the lower chamber as a control group. In all of the assays, the number and proportion of cells were determined by FACS analysis.

### Flow cytometry

Flow cytometry analysis was performed according to standard procedures. Anti-CD11b, anti-CD3, anti-CD19, and anti-CCR6 were stained for peripheral blood cells and neutrophils. Anti-CD3, anti-CD44, and anti-CCR6 were stained for activated T cells. Anti-IL-17, anti-IL-4, anti-IFN-γ, and anti-CCR6 were used for intracellular cytokine staining. Afterward, the cells were analyzed by using BD FACS CantoII or BD FACS Calibur. FACS data were examined using FlowJo (Tree Star, Inc.).

### Intracellular factors staining experiments

First of all, T cells were purified following the above method and stimulated with anti-CD3 (5 μg/mL) and anti-CD28 (3 μg/mL) for 3 days to activate the CD3^+^ T cells. Then, protein transport inhibitors included Ionomycin (500 ng/mL), Phorbol-12-myristate-13-acetate (5 ng/mL) and Brefeldin A (BFA, 5 μg/mL) (Biolegend Cat. No. 420601) were added in the last 4 to 6 h of cell culture activation. The cells were performed by surface antigen staining first, washed with PBS at least two times, resuspended in fixation/permeabilization buffer (BD 554714) for 30 to 60 min, and washed three times with a wash buffer. Afterwards, the cells were stained with the intracellular cytokine antibodies for 90 min, washed three times, followed by FACS analysis.

### Immunohistochemistry and immunofluorescence

The renal tissues were fixed in 4% paraformaldehyde and serially cut after embedding in paraffin. Some of the sections were stained with an antibody directed against the CCL20 (BAF360 R&D) for immunohistochemistry. Each kidney section was observed by light microscopy in a blinded manner. Some tissue sections were stained with the fluorescent antibodies of CD3, CCR6, IL-17, and 4′,6-diamidino-2-phenylindole was used for staining the nucleus. The stained kidney sections were examined and analyzed by a special technical under confocal microscope.

### Statistical analysis

We used Student *t* tests to determine the statistical significance of all experimental data. *P* values less than 0.05 were considered statistically significant.

## Results

### Expression of CCL20 is increased in HMC co-cultured with IgA1 from IgAN

**Fig 1:**
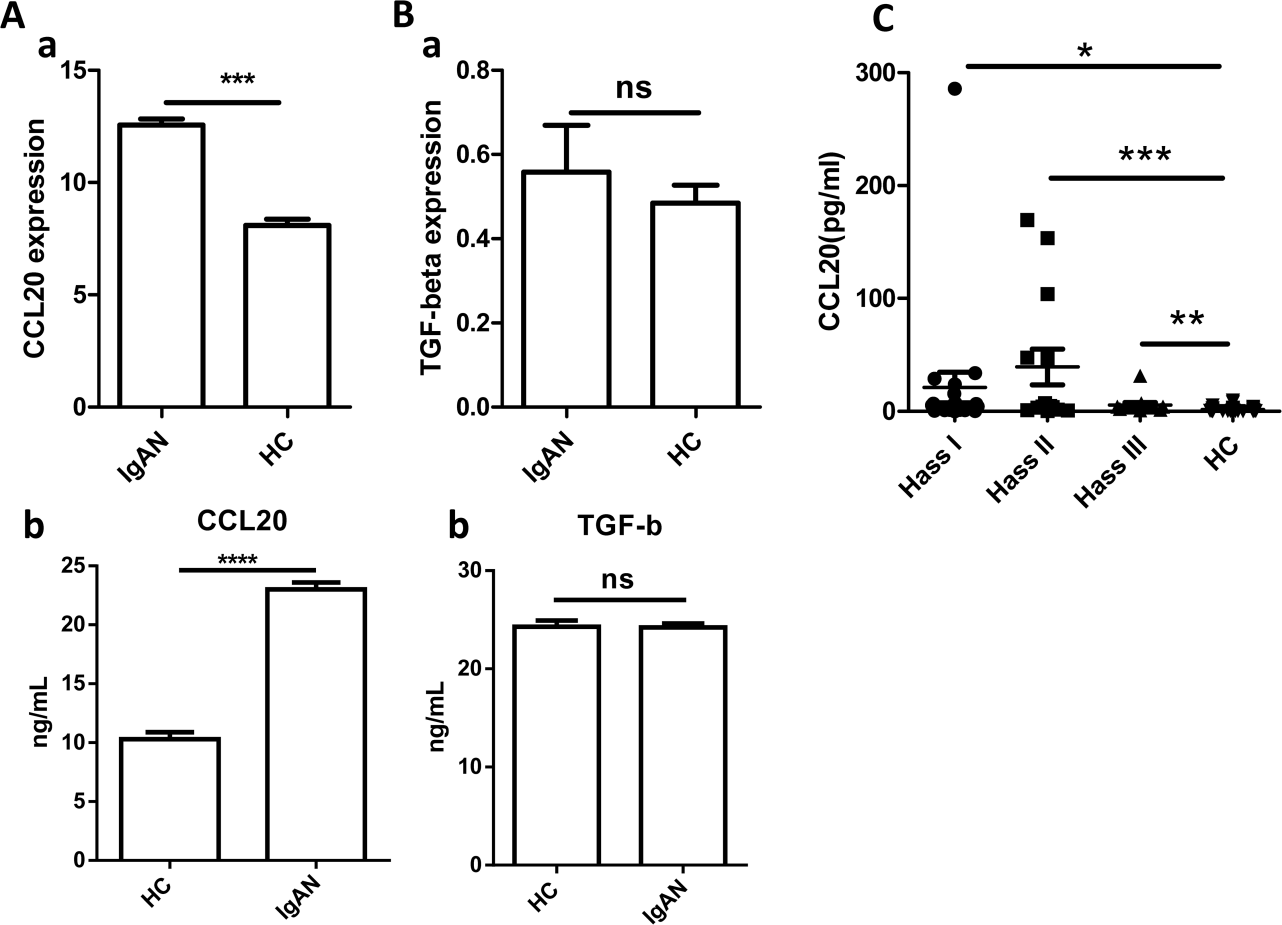
The expression of CCL20 in IgA1-treated HMC and serum from IgAN patients detected by qRT-PCR and ELISA, respectively. (A) mRNA expression level of CCL20(a) and protein level (b) in IgA1 (purified from IgAN patients serum (IgAN) or health control (HC))-treated HMC were evaluated by qRT-PCR (normalized by GAPDH) and ELISA. (B) Meanwhile TGF-beta expression level were also evaluated by qRT-PCR (normalized by GAPDH) (a) and ELISA (b) for IgA1-treated HMC. (C) CCL20 protein levels of serum from different classification of IgAN patients (HassI: *n* = 21, *P* = 0.045. HassII: *n* = 14, *P* = 0.0001. HassIII: *n* = 13, *P* = 0.0049) and HC peoples (*n* = 41) were measured by ELISA. *P<0.05, **P<0.01, ***P<0.001, ns: no significant difference. The data are representative of four independent experiments.

RT-PCR assay was performed to screen the expression of 44 human chemokines of HMC incubated with serum from IgAN or health control (HC) and to determine whether IgAN–IgA1 interaction with HMC can produce chemokines and contribute to the formation of kidney inflammation. The results showed that CCL20 was consistently highly expressed in IgAN serum-treated HMC (data not shown). We purified IgA1 from IgAN and HC serum with Jacalin-Agarose column (InvivoGen, gel-jac-5), respectively, and added the purified IgA1 in the HMC culture system for 24 h, harvested the IgA1-treated HMC, and extracted and subjected the total RNAs to qRT-PCR. The results showed that CCL20 expression in HMC treated with IgAN–IgA1 was significantly higher than that in HMC treated with HC–IgA1 (Fig 1A). However, the TGF-beta expression levels were similar between IgAN–IgA1-treated HMC and HC–IgA1-treated HMC (Fig 1B).

To further confirm whether the protein expression of chemokine CCL20 was indeed higher in IgAN serum, we measured the CCL20 expression level in different degrees of IgAN diseases and HC by ELISA. CCL20 was significantly higher in any kind of IgAN serum than in HC serum (Fig 1C). Therefore, these data suggested that the interaction of IgA1with HMC could induce inflammation by secreting chemokine CCL20.

### CCL20 preferentially attracts CCR6^+^-activated T cells

**Fig 2:**
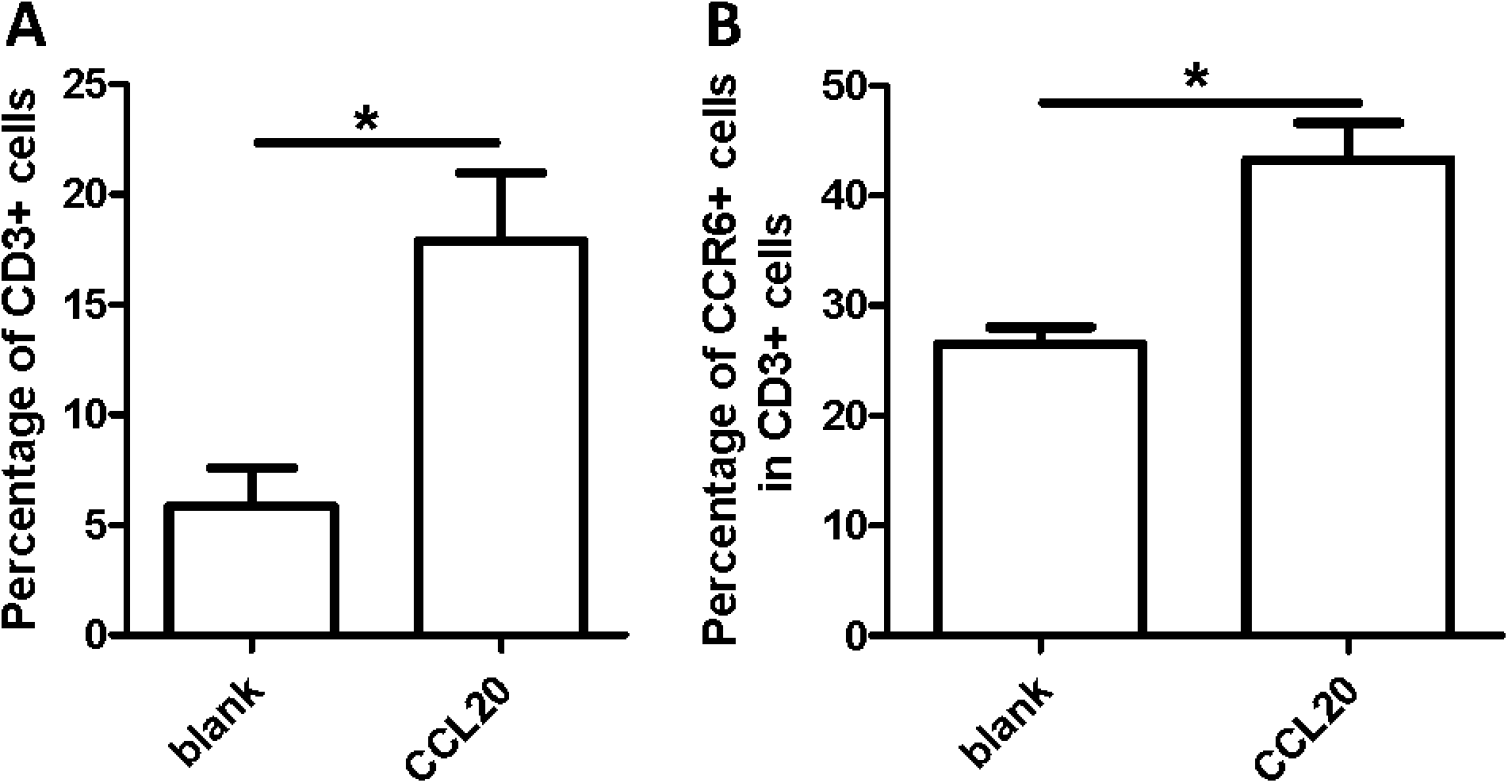
CCL20 mainly attracts CCR6^+^ T cells evaluated by trans-well assay. (A) The attracted cells by CCL20 were more CD3^+^ T cells analyzed by FACS. (B) The attracted T cells by CCL20 were more CCR6^+^ T cells analyzed by FACS. *P<0.05. The data are representative of three independent experiments.

We first performed a chemotaxis assay to test the direct function of CCL20 on peripheral blood immunocytes under normal conditions and to determine which cells are chemoattracted by this higher expression of CCL20 secreted by IgA1-stimulated HMC. We found that the leukocytes migrated toward the bottom of the transwell with recombination-CCL20 in a dose-dependent manner (data not shown). FACS analysis revealed that the percentage of CD3^+^ T cells in the group added with recombination-CCL20 was significantly higher than that in the blank group (Fig 2A). Considering that CCR6 is the only one receptor of CCL20, we further analyzed the percentage of the attracted T cell subsets, with FACS data showing that most of the attracted cells in the addition of CCL20 group were CCR6^+^CD3^+^ T cells (Fig 2B). Therefore, we considered that CCL20 may mainly attract T cells compared with other peripheral immune cells.

**Fig 3:**
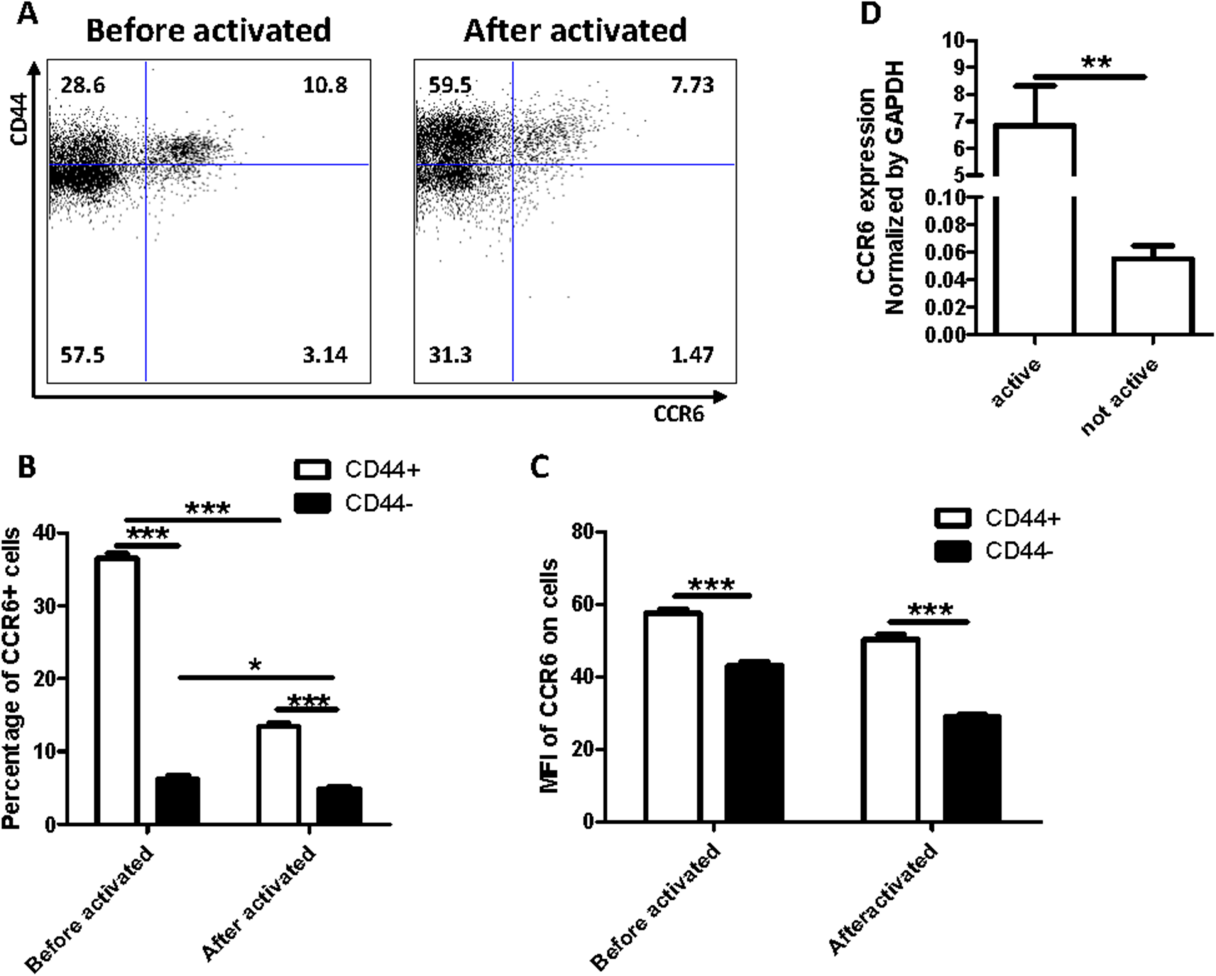
The CCR6^+^ T cells mainly belong to CD44^+^-activated T cells. (A–C) The purified T cells were activated by anti-CD3 and anti-CD28 antibodies and detected CCR6 and CD44 expression by FACS flow cytometry (A). The percentage of CCR6^+^ T cells were calculated according to the FACS analysis (B). The MFI were evaluated by flow Jo software (C). (D) mRNA expression level of CCR6 in activated or non-activated T cells were measured by qRT-PCR (normalized by GAPDH). *P<0.05, **P<0.01, ***P<0.001. The data are representative of three time repeats.

Under disease conditions, large numbers of T cells would be activated under the stimulus of some antigens. CD3^+^ T lymphocytes were purified from PBMC via magnetic bead cell sorting and treated with anti-CD3 (5 μg/mL) and anti-CD28 (3 μg/mL) for 3 days to verify the increase of CCR6 expression in activated T cells. Afterward, the activated T cells were stained with anti-CD3, anti-CD44, and anti-CCR6, followed by FACS analysis. The results showed that most CCR6^+^ T cells were CD44^+^ T cells (Fig 3A and 3B). The mean fluorescence intensity (MFI) of CCR6 was higher in CD44^+^ T cells than in CD44^−^ T cells (Fig 3C).

To verify the experimental results, we extracted the total RNA of the activated and non-activated T cells. RT-PCR was then performed to compare the mRNA expression level of CCR6. Fig 3D shows that the mRNA expression level of CCR6 in the activated T cells was higher than that in non-activated T cells. As the unique receptor of CCL20, CCR6 was mainly expressed in activated T cells.

### CCR6^+^ T cells includes more Th17 cells

**Fig 4:**
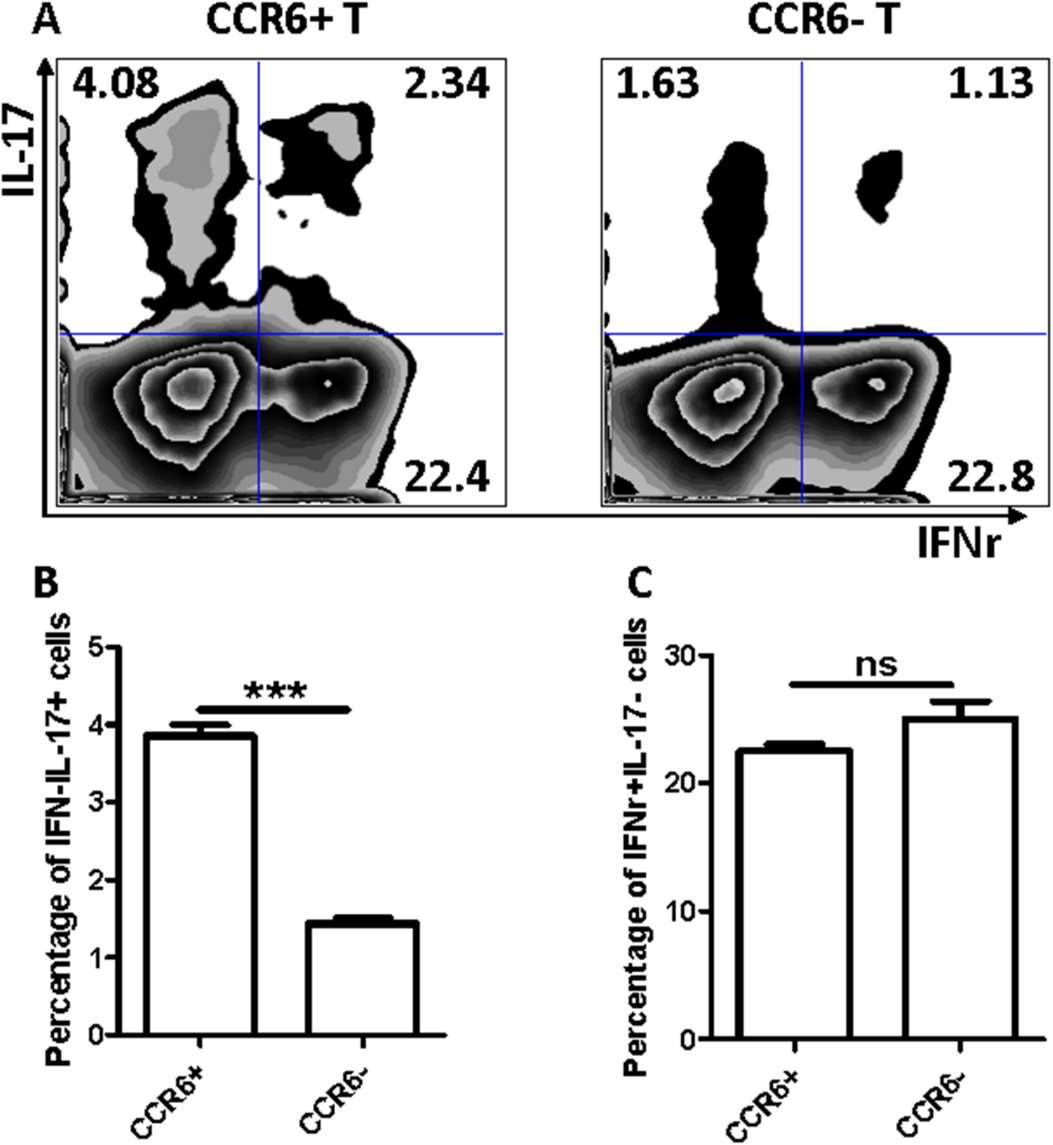
The CCR6^+^ T cells contain more IL-17^+^IFNr^−^ cells. (A) Intracellular staining was conducted to measure the expression of IL-17 and IFNr in CCR6^+^ and CCR6^−^ T cells and analyzed by FACS (B and C). The percentage of IL-17^+^IFNr^−^ (B) and IFNr^+^IL-17^−^ (C) cells were calculated. ***P<0.001, ns: no significant difference. The data are representative of three independent experiments.

T helper cells play a crucial role in orchestrating immune responses by secreting cytokines that regulate various cellular functions [15]. In recent years, IL-17 is perceived as a vital pro-inflammatory cytokine predominantly produced by activating T helper cells, which are also termed Th17 cells [16–18]. Many researchers identified this kind of IL-17-producing Th17 cells also exists in the inflamed kidney [17, 19] and confirmed that Th17 cells perform an immune function against renal diseases [17, 19–21]. The chemokine receptor CCR6 is expressed on the surfaces of Th17 cells in individuals with diseases [22–26]. In Fig 3, CD44^+^ T cells were expectedly increased when T cells were activated with anti-CD3 and anti-CD28 antibodies. By contrast, the CCR6^+^ T cells were not increased. These studies led us to postulate that those CD3^+^CCR6^+^ cells might be IL-17-producing cells (Th17 cells). To confirm this finding, we analyzed the IFN-γ and IL-17A expression in CD3^+^CCR6^+^ T cells compared with those in CD3^+^CCR6^−^ T cells. Intracellular staining and FACS analysis showed that both IFN-γ^−^IL-17^+^ cells were significantly higher in CCR6^+^ cells than that in CCR6^−^ cells; however, IFN-γ^+^IL-17^−^ cells exhibited similar percentage in CCR6^+^ T cells compared to that in CCR6^−^ T cells (Fig 4A–4C), suggesting that CCR6^+^ T cells have more Th17 cells.

**Fig 5:**
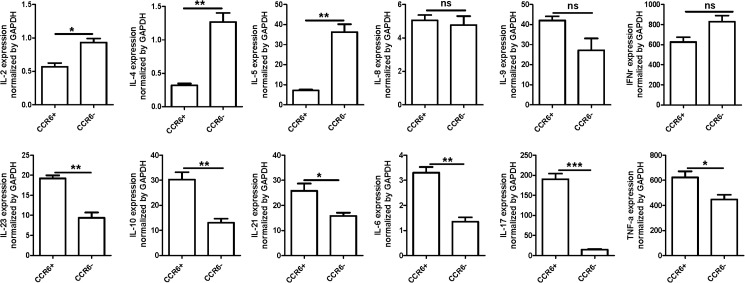
Cytokines expression was detected by qRT-PCR. Total RNA were extracted from CD3+CCR6+ or CD3+CCR6− T cells. The expression of selected cytokines including IL-2, IL-4, IL-5, IL-8, IL-9, TNF-α, IFN-γ, IL-6, IL-10, IL-17, IL-21, and IL-23 were detected by qRT-PCR. *P<0.05, **P<0.01, ***P<0.001. The data are representative of three time repeats.

We further conducted qRT-PCR to quantitate the expression of some cytokines including IL-2, IL-4, IL-5, TNF-α, IFN-γ, IL-6, IL-10, IL-17, IL-21, and IL-23 in CCR6^+^ T cells. Fig 5 showed that TNF-α, IL-6, IL-10, IL-17, IL-21, IL-23, in CD3^+^CCR6^+^ group were significantly higher than the CD3^+^CCR6^−^ group. However, IL-2, IL-4, and IL-5 in CD3^+^CCR6^−^ group were significantly higher than the CD3^+^CCR6^+^ group; no difference was found for the expressions of IFN-γ, IL-8, and IL-9 between CCR6^+^ and CCR6^−^ T cells. Among them, IL-17 expression was extremely higher in CCR6^+^ T cells than that in CCR6^−^ T cells. This alteration of cytokine expression profile either suggested that CCR6^+^ T cells possessed more Th17 cells.

### CCL20 and CCR6^+^IL-17^+^ T cells can be detected in the glomeruli of IgAN patients

**Fig 6:**
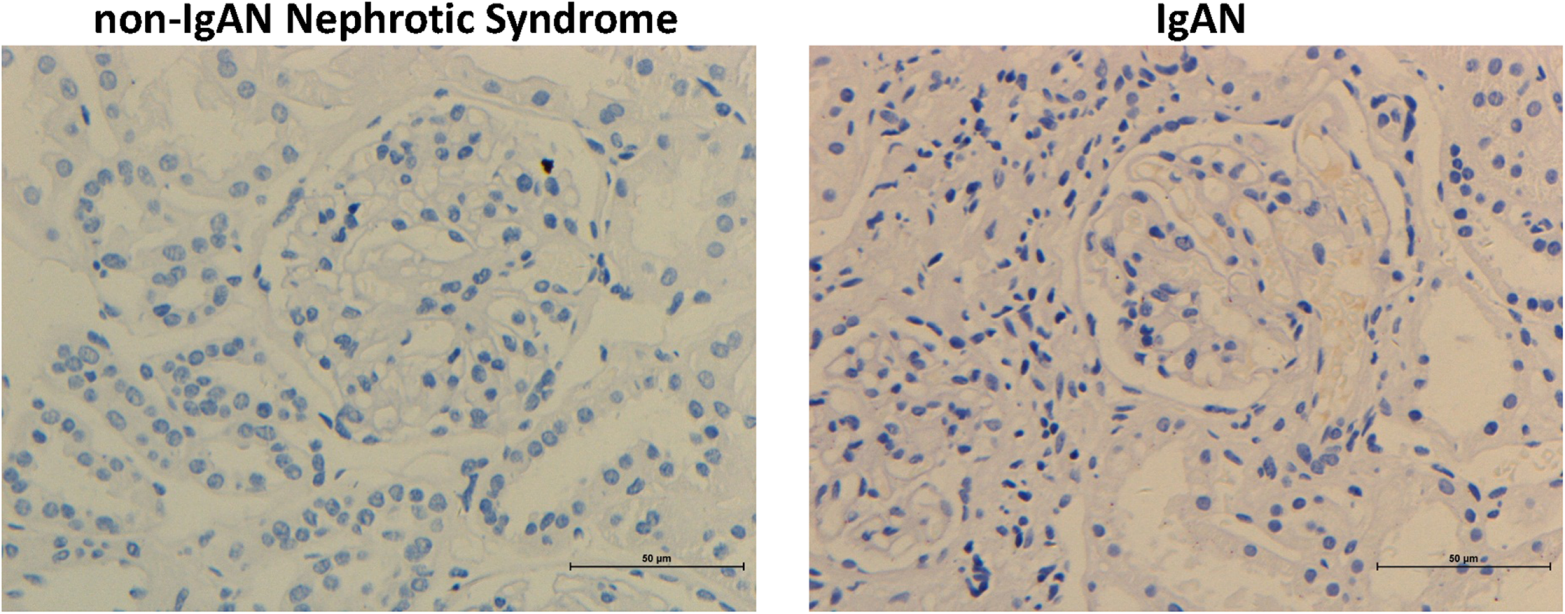
CCL20 are detected by immunohistochemical staining for sections from non-IgAN nephrotic syndrome or IgAN patients.

To confirm that CCL20 is expressed in IgAN in vivo, we examined the glomerulus of patients which were proven to be IgAN via renal biopsy. Immunohistochemical staining was positive for CCL20 in IgAN glomerulus (Fig 6B), but CCL20 was undetectable in normal tissues (Fig 6A).

As previous studies showed, peripheral T cells are activated after antigens are stimulated in IgAN patients, recruited by chemokines to the kidney, and triggered immune inflammatory responses. To confirm that CCR6^+^ T cells were attracted to the kidney in IgAN, we checked the CCR6^+^ T cells in kidney by immunofluorescence staining. Confocal analysis showed that CD3^+^CCR6^+^ indeed existed in IgA kidney tissues but not in normal kidney tissues. This phenomenon suggested that the CCL20 secreted by IgA1-interacted HMC could attract CD3^+^CCR6^+^ T cells to migrate to the kidney.

**Fig 7:**
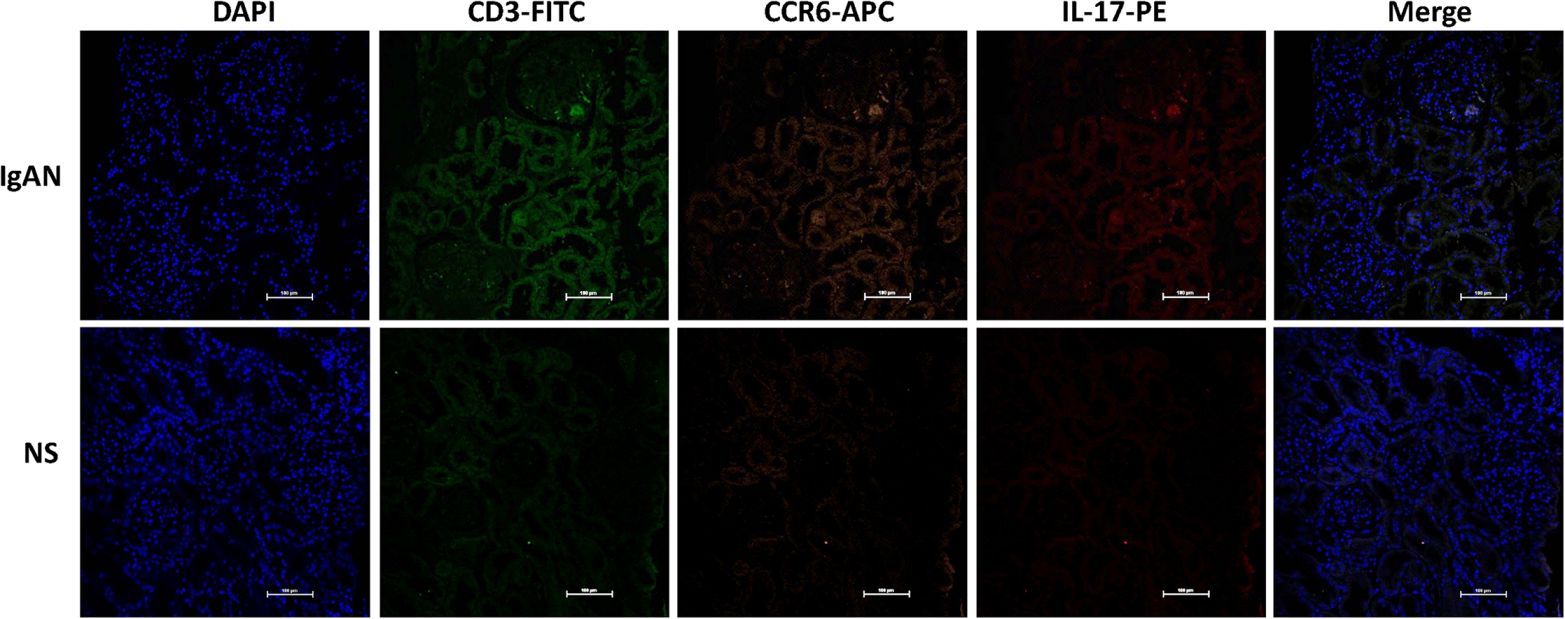
CD3^+^CCR6^+^IL-17^+^ cells are detected by immunofluorescence staining for section from IgAN patients or NS (non-IgAN nephrotic syndrome).

Immunofluorescence staining was performed to confirm that IL-17-produced CCR6^+^ T cells indeed exist in the kidney. The results show that CD3^+^IL-17^+^CCR6^+^ cells existed in IgAN but not in the control kidneys (Fig 7).

Therefore, CCL20 produced by HMC that interacted with IgA1 attracts Th17 cells to the kidneys to induce inflammation and cause further damage.

## Discussion

The aberrant IgA or IgA-related immune complex deposition is an elementary factor in the development of IgAN [10–12]. Immune inflammatory factors, such as CXCL1, MCP-1, TNF-α, IL-1β, IL-6, and IL-8, have been extensively investigated [5, 6, 9, 27]. However, the mechanism by which this IgA1 and IgA1 complex deposition induced inflammation is largely unclear.

The expression and presentation of chemokines in particular microenvironments are involved in the formation of lymphoid tissue and in chronic renal inflammation [28–30]. The chemokines and chemokine receptors are connected with the emergence and development of renal diseases [31]. Therefore, we hypothesized that IgA1 and IgA1 complex may interact with HMC and induce HMC to produce some chemokines followed by attracting inflammation cells to the kidney to cause further damage.

Either serum or IgA1 from IgAN patients can stimulate HMC to alter the expression profile of chemokines. CCL20 is highly overexpressed in serum or IgA1 and HMC incubation system. CCL20, also known as liver-and-activation-regulated chemokine, macrophage inflammatory protein-3α (MIP-3α), or Exodus-1, and its only known receptor, CCR6, which plays a role in the onset and development in various diseases, including tumors, rheumatoid arthritis, pneumococcal meningitis, and human renal inflammation, have been widely explored [32–35]. The CCL20/CCR6 axis is expressed in various tissues by a series of immune cells, including immature effector/memory T-cells, B-cells, and dendritic cells [36, 37]. The mRNA expression of CCR6 is upregulated in T cells as a result of inflammatory stimulation [38]. Th17 cells are important contributors to the pathogenesis of numerous experimental autoimmune diseases, chronic inflammatory diseases, and cancers, such as rheumatoid arthritis, inflammatory bowel disease, glomerulonephritis, and colorectal cancer [22, 35, 36, 39, 40]. In addition, the CCL20 receptor CCR6 is selectively expressed on the surfaces of Th17 cells [15, 23]. Moreover, Th17 cells perform multiple functions by their dominating cytokine, IL-17, which provokes a wide range of immune inflammatory reactions [24] and emerges as a mediator in inflammation-associated diseases [22]. The kidney, as one of the target organs of immune action, is also involved in the immune response of Th17 cells. The pathological damage process of the immune-related diseases is involved in Th17 cells and CCL20 of immunoreaction [39, 41].

Th17 and Th1 cells have been reported to coordinate with each other in the renal inflammatory response [42], mediating inflammation occurrence and development together. Panzer et al. [40] found that it was consistent with this notion in murine crescentic glomerulonephritis. Th17 cells mediated immune response of the renal tissue injury during the early phase, whereas Th1 cells were crucial mediators of the disease at the later stage. Hünemörder [43] also found that Th1 and Th17 cells promote crescent formation in the kidneys in the experimental autoimmune glomerulonephritis model and participate in the progression of renal disease to necrotizing/crescentic GN. The present study demonstrated that HMC-produced CCL20 chemoattracted CCR6^+^ T cells to the kidney, and these CCR6^+^ T cells are mainly Th17 cells. Immunostaining of the kidney tissues of IgAN patients also showed that CCR6^+^IL-17^+^ T cells do exist in IgAN kidney but not in normal kidney tissues. The mRNA expression levels of Th17-related cytokines, including IL-17, IL-21, IL-23, and IL-6, were higher in CCR6^+^ T cells than in CCR6^**−**^ T cells. This finding suggested that Th17 cells may play a dominant role in IgAN.

In summary, this study characterizes the role of CCL20/CCR6 and Th17/IL-17 axes in the pathogenesis of IgAN. Our study showed that CCL20 was produced by HMC incubated with abnormal IgA1 and attracted CCR6^+^ T cells, especially Th17 cells, to injure the kidney. Moreover, these Th17 cells secrete pro-inflammatory and inflammatory factors, especially IL-17. These factors were involved in renal inherent cells, such as mesangial cell surface receptors, and consequently induced a series of inflammatory reactions. As a result, IgAN underwent further development and deterioration. Therefore, our study contributed new information about the mechanisms of IgAN. In particular, targeting the CCL20/CCR6 and Th17/IL-17 axis antagonists may be used for new therapeutic strategies to prevent disease progression and treat IgAN. However, the detailed molecular mechanisms of IgA1 interacting with HMC to induce CCL20 expression are not presented. The attraction of CCR6+ Th17 cells by CCL20 plays a dominant role in IgAN inflammation and is currently awaiting the verification of the animal model. Transferrin receptor 1 (CD71 or TFR1) was also found to be a receptor for IgA. CD71 protein is overexpressed in human IgAN biopsies and is colocalized with IgA mesangial deposition. In vitro studies have shown that polymeric IgA1 interactions with CD71 on human mesangial cells induce cell proliferation and secretion of IL-6 through activation of phosphoinositide 3-kinase (PI3K)/protein kinase B (AKT)/mechanistic target of rapamycin and the extracellular signal-regulated kinase (ERK) 1/2 kinase pathways [44]. Future studies should determine whether the interaction of IgA1 with HMC can induce CCL20 expression and whether this phenomenon is due to the CD71-PI3K/AKT/ERK pathway.
